# Redox-Responsive Coordination Polymers of Dopamine-Modified Hyaluronic Acid with Copper and 6-Mercaptopurine for Targeted Drug Delivery and Improvement of Anticancer Activity against Cancer Cells

**DOI:** 10.3390/polym12051132

**Published:** 2020-05-14

**Authors:** Bo Tao, Zongning Yin

**Affiliations:** Key Laboratory of Drug Targeting and Drug Delivery Systems, West China School of Pharmacy, Sichuan University, No. 17, Block 3, Southern Renmin Road, Chengdu 610041, China; apharmacy@163.com

**Keywords:** coordination, hyaluronic acid, 6-Mercaptopurine, redox-responsive, targeting

## Abstract

Dopamine-modified hyaluronic acid (HA-DOP) was chosen as the drug carrier in this study, and Cu^2+^ was selected from among Cu^2+^, Zn^2+^, Fe^2+^, and Ca^2+^ as the central atom. 6-Mercaptopurine (6-MP) was conjugated with HA through a coordination reaction. HA-DOP-copper-MP (HA-DOP-Cu-MP), a redox-responsive coordination polymer prodrug, was prepared. The drug loading was 49.5 mg/g, the encapsulation efficiency was 70.18%, and the particle size was 173.5 nm. HA-DOP-Cu-MP released rapidly in the release medium containing reduced glutathione (GSH), and the accumulated release exceeded 94% in 2 h. In the release medium without GSH, the drug release rate was slow, with only 15% of the 6-MP released in 24 h. Cell uptake experiments revealed the CD44 targeting of HA. Cell viability assays showed that the cytotoxicity of HA-DOP-Cu-MP was higher than that of free 6-MP. Indeed, HA-DOP-Cu-MP is very toxic to cancer cells. In this paper, the redox-responsive drug delivery system was synthesized by a coordination reaction. The tumour targeting and tumour cytotoxicity of 6-MP were improved.

## 1. Introduction

Cancer is the primary cause of morbidity and mortality worldwide. The pharmaceutical industry has invented many cytotoxic drugs that could potentially cure cancer. Unfortunately, these drugs also have enormous systemic toxicity to normal tissue. Polymers and polymeric microparticles drug delivery systems can improve solubility, prolong drug release time, and enhance targeting of many drugs [1,2,3,4,5,6]. The new generation of multifunctional nanodrug delivery systems can release drugs after stimulation, such as pH, temperature, redox, magnetic or electrical field, light, pressure, ultrasound, enzymes, and glucose stimulation [7,8].

Most stimuli-responsive carriers release drugs by breaking chemical bonds, such as hydrazine [9], oxime [10], azo [11], or disulfide bonds [12]. Recently, researchers found that drugs could also be released by breaking coordination bonds. The relevant complexes are composed of central atoms (M, metal) and ligands (L, ligand). Coordination chemistry constitutes the bridge between organic chemistry and inorganic chemistry. Complexes have many bonding modes and structural types. A variety of materials with special functions can be synthesized through coordination reactions, such as conductive functional complexes, luminescent functional complexes, magnetic functional complexes, and complex porous materials. Gd0^3+^, Fe^3+^, and Mn^2+^ complexes can be used as contrast agents for nuclear magnetic resonance (NMR) imaging in medicine [13]. The cisplatin (PtCl_2_ (NH_3_) _2_) metal complex is used to treat cancer [14]. The complexation of a drug and a metal improves the water solubility of insoluble drugs [15]. Additionally, the use of such complexes reduces the toxic side effects and releases the drugs only under certain conditions, such as low pH [16], enzymes [17], redox [18], and light [19]. Indeed, complex prodrugs synthesized from metals and drugs have attractive prospects [20,21].

The coordination ability of the groups of many delivery vehicles is weak, making it difficult to form complexes with drugs. Mussels provide a solution to this problem. Mussels can adhere to reefs, ships, and even paraffin and Teflon. Mussels secrete a type of adhesive protein that contains dopamine, which is the key to mussels’ superior adhesive capacity [22]. Dopamine is widely distributed in the human body and is an important neurotransmitter [23]. The two phenolic hydroxyl groups of dopamine form stable complexes with some metal ions. The strength of the bonds between dopamine and metal ions (0.8 nN) is similar to the typical strength of a covalent bond (2 nN) [24]. This characteristic is why dopamine is widely used to modify polymers for drug delivery, such as poly (ethylene glycol) (PEG), chitosan, mesoporous silica, and hyaluronic acid (HA). Indeed, dopamine is an ideal ligand.

HA is an anionic linear polysaccharide consisting of repeats of two sugar units: D-glucuronic acid and *N*-acetyl glucosamine [25,26]. HA is a major component of the extracellular matrix and can bind to receptors, such as hyaluronan-mediated motility receptor (RHAMM), CD44, lymphatic vessel endothelial hyaluronan receptor 1 (LYVE-1), liver-expressed chemokine (LEC), Toll-like receptor 4 (TLR4), hyaluronan receptor for endocytosis (HARE), and tumour necrosis factor-inducible gene 6 (TSG6) [27,28]. Most tumour cells, such as liver cancer, breast cancer, melanoma, and colon cancer cells, highly express the CD44 receptor. The CD44 receptor can bind to HA oligosaccharide units with as few as four monosaccharides [29], indicating that HA has a strong ability to bind to CD44. Therefore, HA is an excellent tumour-targeting vector and is widely used in polymer drug delivery systems [30].

6-Mercaptopurine (6-MP) is a purine antagonist chemotherapeutic drug and immunosuppressive agent. It is usually used to treat a variety of diseases, such as acute lymphoblastic leukaemia, malignant lymphoma, multiple myeloma, chorionic epithelial cell carcinoma, rheumatoid arthritis, and enteritis. It is also used to prevent rejection after organ transplantation [31,32,33]. The solubility of 6-MP in water is low, and this compound is only soluble in sodium hydroxide solution or organic solvents, such as dimethyl sulfoxide (DMSO). Free thiol readily combines with plasma protein to form disulfide bonds. 6-MP has the shortcomings of systemic toxic side effects, a short half-life, low bioavailability, and gastrointestinal reactions after oral administration [34].

**Figure 1:**
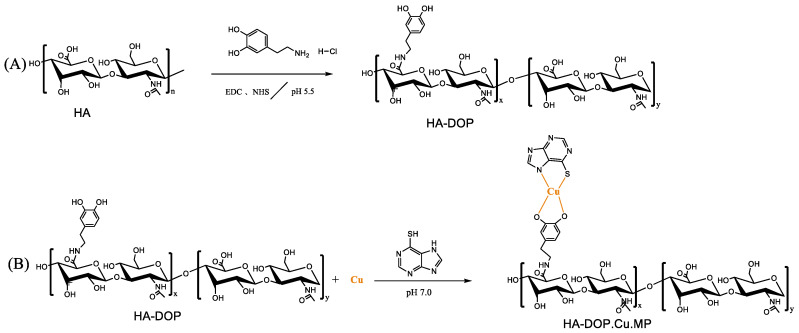
(**A**) Synthesis scheme of dopamine-modified hyaluronic acid (HA-DOP) via 1-(3-Dimethylaminopropyl)-3-ethylcarbodiimide hydrochloride/*N*-hydroxysuccinimide (EDC/NHS) coupling chemistry. (**B**) Synthesis scheme of HA-DOP-Cu-MP coordination polymers via a coordination reaction.

**Figure 2:**
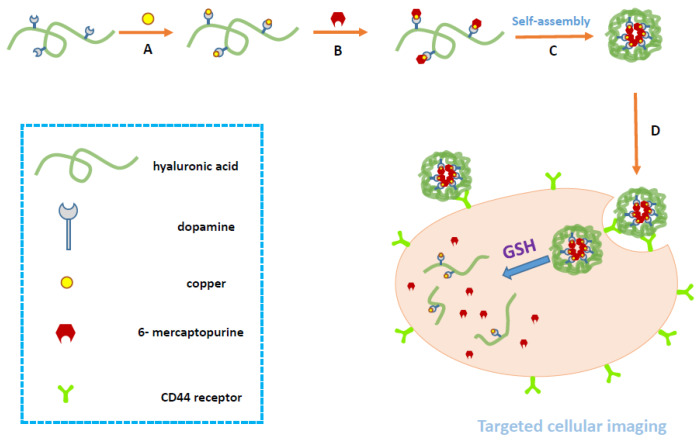
Schematic illustration of the synthesis, self-assembly, and intracellular trafficking pathway of HA-DOP-Cu-MP coordination polymer nanoparticles. The intracellular trafficking pathway includes receptor-mediated endocytosis, reduction-triggered coordination polymer nanoparticle disassembly, and drug release. GSH, glutathione.

To avoid these shortcomings and increase the water solubility and efficacy of 6-MP, we chose HA as the carrier and modified it with dopamine to enhance its coordination ability. HA-dopamine-copper-MP (HA-DOP-Cu-MP) was synthesized via a coordination reaction (Figure 1). Redox reactions are ubiquitous in cells and play an important role in the process of cell metabolism. Reactive oxygen species (ROS) such as H_2_O_2_ and NO can impair normal cell function. When the concentration of ROS in the cell is too high, in order to react with too much ROS, 5 to 10 mM glutathione (GSH) is usually present in healthy cells [35]. Compared with normal cells, cancer cells exhibit sustained metabolic oxidative stress, mainly owing to inherent mitochondrial dysfunction and NOX activation. As part of metabolic reactions, high levels of ROS are produced [36], so the content of glutathione in tumor cells is higher. For example, the GSH concentration in A549 cells is seven times higher than that in healthy cells [37,38]. Thus, intracellular GSH can be used as a stimulating signal. Blood vessels in tumor tissue are more permeable than healthy tissue, and nanoparticles can penetrate through these gaps. In addition, there is no lymphatic drainage in tumor tissue, and nanoparticles can selectively accumulate in tumor [39,40]. When the HA-DOP-Cu-MP coordination polymer was internalized by tumour cells, 6-MP was released in the presence of GSH, killing the tumour cells (Figure 2).

## 2. Experimental

### 2.1. Materials

Sodium hyaluronate (molecular weight: 5 kDa) was purchased from Bloomage Freda Biopharm Co. Ltd. (Jinan, Shandong, China). 1-(3-Dimethylaminopropyl)-3-ethylcarbodiimide hydrochloride (EDC), *N*-hydroxysuccinimide (NHS), cystamine hydrochloride, fluorescein isothiocyanate isomer I (FITC), glutathione, and 6-MP were purchased from Aladdin Chemical Reagent Co., Ltd. (Shanghai, China). Cystamine dihydrochloride, dopamine hydrochloride, and 1,6-hexanediamine dihydrochloride were purchased from Bide Pharmatech Co., Ltd. (Shanghai, China). The A549 cell line was a generous gift from the Key Laboratory of Drug Targeting and Drug Delivery Systems (West China School of Pharmacy, Sichuan University, Chengdu, China). The NIH/3T3 cell line was kindly provided by Stem Cell Bank (Chinese Academy of Sciences, Shanghai, China). The RPMI1640 medium and penicillin-streptomycin liquid were purchased from HyClone Co. Ltd. (South Logan, UT, USA). Dulbecco’s modified Eagle’s medium (DMEM) was purchased from GIBCO. (Waltham, MA, USA). Foetal bovine serum (FBS) was purchased from FMG Biotech Co. Ltd. (ShangHai, China). 3-(4,5-Dimethylthiazol-2-yl)-2,5-diphenyltetrazolium bromide (MTT) was purchased from Amresco, (Solon, OH, USA). and pancreatin was purchased from Sigma-Aldrich (Shanghai, China).

### 2.2. Methods

#### 2.2.1. Synthesis of HA-DOP

HA (0.1 g) was dissolved in 8 mL of 4-morpholineethanesulfonic acid (MES, pH 6.0) buffer solution, and the pH was adjusted to 4.7. EDC (0.24 g) and NHS (0.12 g) were dissolved in the solution, and the pH was adjusted to 5.7. After incubation for 15 min, dopamine hydrochloride (0.47 g) was dissolved in the reaction solution and mixed under magnetic stirring for 3 h. The reaction mixture was purified by dialysis (molecular weight cut-off (MWCO): 3000) against deionized water for 12 h. The dialysis treatment was repeated four times. Finally, HA-DOP was isolated by freeze-drying and kept in a desiccator until use.

#### 2.2.2. Synthesis of HA-1,6-Hexanediamine dihydrochloride (HA-HDA)

HA (0.3 g) was dissolved in 24 mL of MES (pH 6.0). After stirring for 2 h, the pH was adjusted to 4.7. EDC (0.72 g) and NHS (0.35 g) were dissolved in the solution, and the pH was adjusted to 5.7. After incubation for 15 min, 2.84 g of HDA was dissolved in the reaction system and mixed under magnetic stirring for 5 h. The reaction mixture was purified by dialysis (MWCO: 3000) against deionized water for 12 h. The dialysis treatment was repeated four times. Finally, HA-HDA was isolated by freeze-drying and kept in a desiccator until use.

#### 2.2.3. Study of the Coordinating Abilities of Cu^2+^, Zn^2+^, Fe^2+^, and Ca^2+^ with 6-MP

Stock solutions (50 mM) of Cu^2+^, Zn^2+^, Fe^3+^, and Ca^2+^ were prepared by dissolving Cu(NO_3_)_2_·3H_2_O, ZnCl_2_, FeSO_4_·7H_2_O, and CaSO_4_ in water, respectively. Then, the 6-MP solution (50 mM) was dispersed in each stock solution to observe whether any precipitate was generated.

#### 2.2.4. Study of the Stability of HA Derivatives Coordinated with Cu^2+^ and 6-MP

The reaction solution (5 mg/mL) was prepared by dissolving HA, HA-HDA, or HA-DOP in water. A solution of Cu(NO_3_)_2_ in ethanol was added to the reaction solution, and the pH was adjusted to 7.0. Then, 6-MP in ethanol (1 mg/mL) was added to the reaction solution to observe whether precipitation appeared after standing for 12 h.

### 2.3. Drug Loading, Entrapment Efficiency, and Solubilization Properties of HA-DOP-Cu-MP

**6-MP content in HA-DOP-Cu-MP.** HA-DOP (200 mg) was dissolved in 40 mL water. A solution of Cu(NO_3_)_2_ (24 mg Cu(NO3)_2_·3H_2_O, dissolved in 12 mL ethanol solution) was added to the reaction solution, and the pH was adjusted to 7.0. Then, a solution of 6-MP (17 mg 6-MP.H_2_O, dissolved in 17 mL ethanol solution) was added to the HA-DOP solution, followed by a 12 h incubation at 23 °C. The resulting conjugate was dialyzed seven times against deionized water, and HA-DOP-Cu-MP was obtained by freeze-drying. HA-DOP-Cu-MP (5 mg) was dissolved in water (10 mL) to generate the mother liquor. Potassium dihydrogen phosphate buffer (0.35 mL, pH 7.4), potassium permanganate solution (1.5 mL, 1 mM), NaOH solution (0.8 mL, 1 M), and deionized water (2.2 mL) were added sequentially to 0.15 mL of the mother liquor. After incubation for 48 h, the fluorescence signal of the final reaction mixture was monitored. The excitation wavelength was 286 nm, and the emission wavelength was 397 nm. The content of 6-MP in the solution was calculated according to the fluorescence intensity. The drug loading and entrapment efficiency were calculated based on the content of 6-MP.

$$\mathrm{Drug}\;\mathrm{Loading}\;\left(\mathrm{DL}\right)=\frac{\mathrm{The}\;\mathrm{content}\;\mathrm{of}\;6-\mathrm{MP}\;\mathrm{in}\;\mathrm{HA}-\mathrm{DOP}.\mathrm{Cu}.\mathrm{MP}}{\mathrm{The}\;\mathrm{weight}\;\mathrm{of}\;\mathrm{HA}-\mathrm{DOP}.\mathrm{Cu}.\mathrm{MP}}\times 100\%\;\mathrm{Entrapment}\;\mathrm{Efficiency}\;\left(\mathrm{EE}\right)=\frac{\mathrm{Actual}\;\mathrm{drug}\;\mathrm{loading}}{\mathrm{Theoretical}\;\mathrm{drug}\;\mathrm{loading}}\times 100\%$$

**Determination of the solubility of 6-MP in aqueous solution**. 6-MP (3.0 mg) was dissolved in water (3 mL, 37 °C) at a rotating speed of 100 r/min. After being held in a water bath for 72 h and then centrifuged at 10,000 rpm for 3 min, the supernatant was diluted and measured at 323 nm on a UV-vis spectrophotometer.

**Study of the solubilization of 6-MP by the coordination polymer**. HA-DOP-Cu-MP was dissolved in water. Then, the solubility of 6-MP was calculated based on the amount of HA-DOP-Cu-MP in the water.

### 2.4. Characterization of HA-DOP-Cu-MP

#### 2.4.1. Particle Size Measurements of HA-DOP-Cu-MP

HA-DOP-Cu-MP was dissolved in phosphate-buffered saline (PBS) (pH 7.4), and the particle size was measured by dynamic light scattering (Zetasizer Nano ZS90, Malvern, Malvern, UK).

#### 2.4.2. Characterization of the ^1^H-NMR Spectrum

The sample was dissolved in 0.5 mL of deuterium oxide. The ^1^H-NMR spectra were recorded on a 400 MHz NMR spectrometer (Bruker AVANCE III 400 NMR spectrometer, Bruker, Billerica, MA, USA).

#### 2.4.3. Fourier Transform Infrared (FT-IR) Spectroscopy

HA-DOP-Cu-MP or 6-MP was ground with KBr and pressed to form disks, which were then analysed by FT-IR spectroscopy (Nicolet MAG-560, Thermo, Waltham, MA, USA).

#### 2.4.4. X-Ray Diffraction (XRD) Spectroscopy

The XRD spectra of HA-DOP-Cu-MP and 6-MP.H_2_O were collected using an X-ray diffractometer (EMPYREAN Panalytical, Almelo, Netherlands). The diffraction angle (2*θ*) was recorded from 5° to 60° with a step width of 0.025°, and Cu was used as the source of X-ray radiation at 40 kV.

### 2.5. Drug Release Experiment

The in vitro drug release rate of MP from HA-DOP-Cu-MP was investigated using a dialysis method. The HA-DOP-Cu-MP (7 mg) was dissolved in 5 mL of potassium phosphate buffer (pH 7.4) and packaged in a dialysis tube (MWCO: 3000). The sealed dialysis tube was placed in potassium phosphate buffer (pH 7.4) with or without 10 mM GSH. The release experiment was performed at 37 °C in a water bath at a rotating speed of 50 r/min. At appropriate time intervals, 0.5 mL of the release media was withdrawn. Then, 0.5 mL of the fresh release medium was added back to the system to maintain the total volume. The cumulative release was calculated based on the test results.

**Determination method.** Potassium permanganate solution, NaOH solution, and deionized water were added sequentially to the sample. After incubation for 48 h, the fluorescence signal of the final reaction mixture was monitored (RF-5301PC, SHIMADZU, Kyoto, Japan). The excitation wavelength was 286 nm, and the emission wavelength was 397 nm.

### 2.6. Cell Uptake Assay

FITC-labelled HA (HA-FITC) was prepared for the cell uptake assay. HA (0.1 g) was dissolved in 8 mL of MES solution (0.1 M). The pH was adjusted with 1 M HCl to 4.7, EDC (0.24 g) and NHS (0.12 g) were dissolved in the solution, and the solution was mixed under magnetic stirring for 15 min. Cystamine dihydrochloride (0.56 g) was added, and the pH of the solution was adjusted to 5.5. After allowing the conjugation reaction to proceed for 3 h, the reaction mixture was purified by successive dialysis (MWCO: 3000) against deionized water for 12 h. The dialysis treatment was repeated four times. Finally, HA-cystamine was isolated after freeze-drying. Two millilitres of FITC in DMSO (6.5 mg/mL) was added to a sodium bicarbonate solution (pH 9.3) of HA-cystamine (30 mg/3 mL), and the mixture was stirred for 2 h at room temperature. The reaction mixture was dialyzed against deionized water three times (3 h each time). Finally, the mixture was precipitated into anhydrous ethanol and washed twice with anhydrous ethanol. HA-FITC was obtained after the precipitate was dried at 40 °C.

A549 cells (cultured in RPMI1640 supplemented with 10% FBS, 1% penicillin, and 1% streptomycin) and NIH/3T3 cells (cultured in DMEM supplemented with 10% FBS, 1% penicillin, and 1% streptomycin) were seeded in 12-well plates (2 × 10^6^/well) and incubated for 24 h at 37 °C. Then, the culture medium was replaced with 1 mL of serum-free culture medium containing HA-FITC (the concentration of FITC was 2.5 μg/mL) and incubated for 4 h at 37 °C. To test whether HA-FITC uptake was mediated by the CD44 receptor, 2.5 mg/mL free HA was added to one group of A549 cells and incubated for 2 h before the addition of HA-FITC.

After incubation with HA-FITC for 4 h, the cells were washed three times with PBS (pH 7.4) and then trypsinized. The cells were blown by pipet to form a cell suspension in complete culture medium and then centrifuged. Finally, the cells were suspended in 0.35 mL of PBS, and the fluorescence intensity of the cells was monitored by flow cytometry (BECKMAN FC500, Brea, CA, USA).

### 2.7. Cell Viability Assays

The cytotoxicity of HA-DOP-Cu-MP and 6-MP against A549 cells was evaluated using the MTT assay. Different concentrations of HA-SS-MP and 6-MP solutions were prepared. The cells were seeded in 96-well plates at a density of 4000 cells per well. After incubation for 24 h, serum-free RPMI1640 culture medium containing different concentrations of HA-DOP-Cu-MP or 6-MP (0.1, 1, 10, and 100 μg/mL) was added to displace the old cell culture medium. After incubation for 48 h, 100 μL of RPMI1640 culture medium containing MTT was added to the cell culture medium and incubated for another 4 h. Then, the RPMI1640 culture medium was removed, and 150 μL of DMSO was added. Finally, the optical absorbance of the solution in each well was measured at 490 nm (iMark™ Microplate, Bio-Rad, Hercules, CA, USA), and the cell viability was calculated based on the absorbance.

## 3. Results and Discussion

### 3.1. Synthetic Method

#### 3.1.1. Study of the Coordination Abilities of Cu^2+^, Zn^2+^, Fe^2+^, and Ca^2+^ with 6-MP

**Figure 3:**
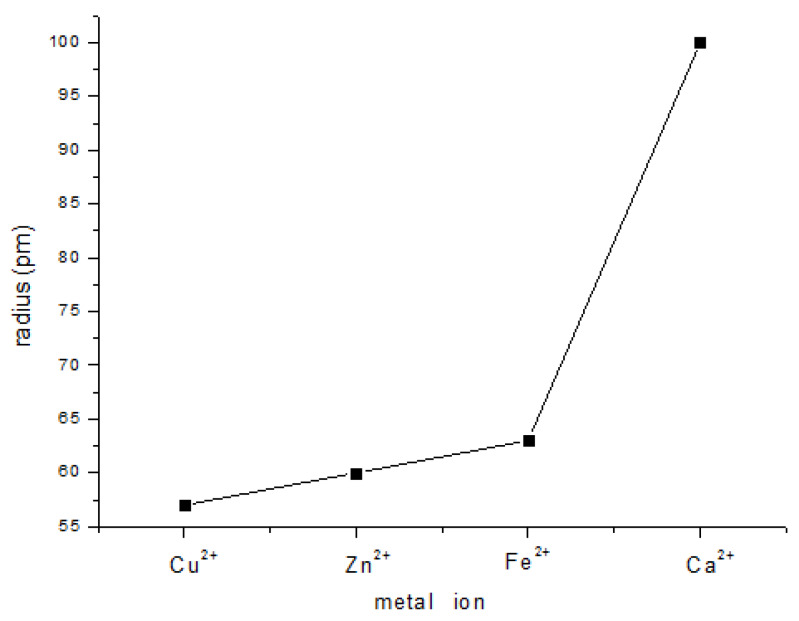
Plot showing the metal ion radii of Cu^2+^, Zn^2+^, Fe^2+^, and Ca^2+^.

Most metal ions, especially transition metal ions, can be used as central atoms in complexes. The coordination abilities of 6-MP with Cu^2+^, Zn^2+^, Fe^2+^, and Ca^2+^ in aqueous solution were investigated and judged by whether a precipitate formed in aqueous solution. Cu^2+^ coordinated with 6-MP resulted in the formation of a brown precipitate in aqueous solution. In contrast, no precipitate was observed for Zn^2+^, Fe^2+^, and Ca^2+^. These experimental results implied that, among the metal ions tested, Cu^2+^ had the strongest coordination ability with 6-MP; therefore, Cu^2+^ was chosen as the central atom. The radius of the central atom strongly influences the stability of the resulting complex. The ionic radius of Cu^2+^ is the smallest among the metals tested (Figure 3), and as a result, the complex formed by Cu^2+^ is the most stable in aqueous solution.

#### 3.1.2. Study of the Stability of HA Derivatives Coordinated with Cu^2+^ and 6-MP

Generally, ligand atoms are electronegative non-metal elements, and ligand groups are composed of elements such as F, Cl, N, O, and S. HA contains carboxyl groups, which can form complexes with certain metal ions under specific conditions. In this paper, HA was modified by hexanediamine or dopamine, and the stability of modified or unmodified HA coordinated with copper ions and 6-MP was studied. Initially, the solutions were clear and transparent, indicating that HA, HA-HDA, and HA-DOP formed complexes with Cu ions and 6-MP. After 12 h, HA-Cu-MP and HA-HDA-Cu-MP formed precipitates, indicating that the complexes formed via carboxyl and amino groups were unstable. In contrast, no precipitation was observed in the aqueous solution of HA-DOP-Cu-MP, indicating that the coordination ability of dopamine-modified HA is relatively strong. The HA-DOP-Cu-MP complexes dissolved and dispersed in aqueous solution in a stable manner. The simplest complex is formed by a monodentate ligand reacting with a central ion. The chelate is a cyclic structure complex, which is formed by a polydentate ligand and a central ion. Because of the ring structure, a chelate is more stable than a simple complex [41]. Amino groups and carboxyl groups form simple complexes with copper ions. The two ionized phenolic hydroxyl groups of the dopamine form bidentate complexes with copper ions, which were more stable than the simple complexes. Therefore, dopamine was selected to modify HA and enhance its coordination ability.

### 3.2. Drug Loading, Entrapment Efficiency, and Solubilization Properties of HA-DOP-Cu-MP

The drug loading of HA-DOP-Cu-MP was 49.5 mg/g, and the encapsulation efficiency was 70.18%. The solubility of 6-MP was 336 μg/mL in aqueous solution. The solubility of the MP in HA-DOP-Cu-MP exceeded 3812 μg/mL in aqueous solution. Compared with the solubility of free 6-MP, that of HA-DOP-Cu-MP was more than 10 times higher. Thus, the coordination polymer greatly improved the solubility of 6-MP. Therefore, this drug delivery system represents a new method for preparing 6-MP injections.

### 3.3. Characterization of HA-DOP-Cu-MP

#### 3.3.1. Particle size of HA-DOP-Cu-MP

The HA-DOP-Cu-MP coordination polymer consists of hydrophilic HA and hydrophobic 6-MP. In aqueous solution, HA forms a hydrophilic shell and 6-MP forms a hydrophobic core. The particle size of HA-DOP-Cu was 319.7 nm and the particle size of HA-DOP-Cu-MP was 173.5 nm (Table 1). The complexes formed by HA-DOP-Cu and 6-MP were further compressed by the hydrophobic interactions of 6-MP, and as a result, the particle size decreased compared with HA-DOP-Cu. In many solid tumours, the vascular endothelial cell tissue will form pores with diameters of 200 nm to 1.2 μm because of its rapid growth [42]. In addition, the lymphatic drainage is impaired. Therefore, HA-DOP-Cu-MP nanoparticles can flow through the tumour vessels in the blood circulation and accumulate in the loose tumour tissues because of the enhanced permeability and retention (EPR) effect [43].

#### 3.3.2. Characterization of the ^1^H-NMR Spectrum

The difference between the ^1^H-NMR spectra of the complex and the organic compounds is caused by the metal ions in the complex. The impact of different metal ions on the ^1^H-NMR spectra of complexes is different. Depending on whether a net magnetic moment is present, metal ions are classified into paramagnetic metal ions and anti-magnetic metal ions. The impact of diamagnetic metal ions on the ^1^H-NMR spectra of complexes is not significant, while paramagnetic metal ions strongly affect the ^1^H-NMR spectra of complexes owing to the existence of a magnetic field. As a result, the ligand of a complex is not detected. These changes can be used to determine whether the metal ion is coordinated with the ligand.

**Figure 4:**
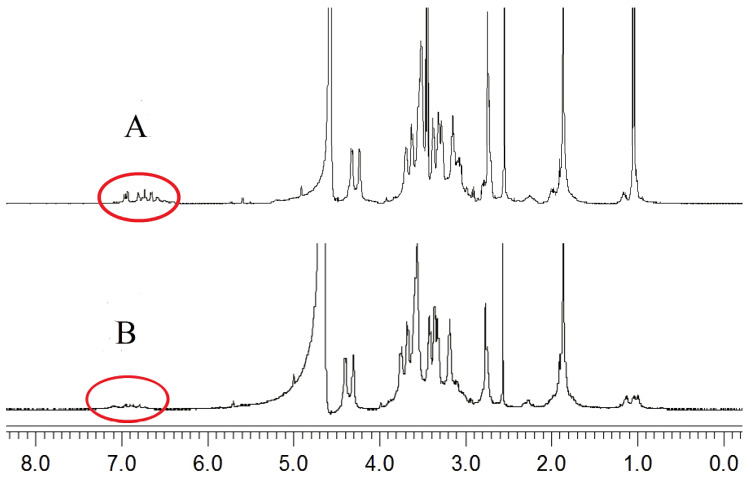
^1^H-NMR spectra of (**A**) HA-DOP and (**B**) HA-DOP-Cu-MP.

The characteristic peaks at 6.79–7.12 ppm were assigned to the protons of the dopamine moiety (Figure 4A) [44,45]. After the phenolic hydroxyl groups on the dopamine formed a complex with the metal ions, the protons on the ligands were replaced by metal ions, forming lone pair electrons. Cu^2+^, which contains unpaired electrons, is a paramagnetic metal ion [46]. Under the influence of Cu^2+^, the ^1^H-NMR peaks of the dopamine groups disappeared (Figure 4B) [47]. Therefore, Cu^2+^, 6-MP, and HA-DOP formed complexes.

#### 3.3.3. FT-IR Spectroscopy Measurement

**Figure 5:**
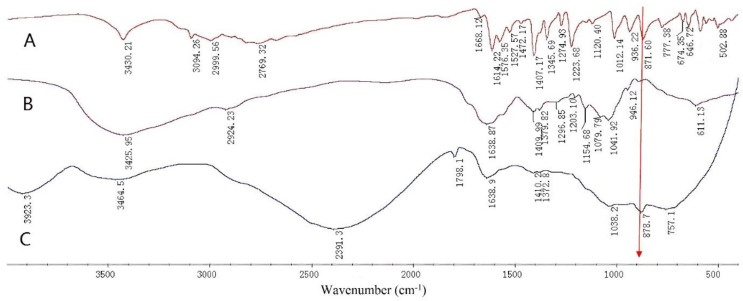
Fourier transform infrared (FT-IR) spectra of (**A**) 6-mercaptopurine (6-MP), (**B**) HA-DOP, and (**C**) HA-DOP-Cu-MP.

The FT-IR spectra collected over the spectral range of 400–4000 cm^−1^ for 6-MP, HA-DOP, and HA-DOP-Cu-MP are presented in Figure 5.

The band at 3425.95 cm^−1^ is assigned to the O-H of HA. The band at 2924.23 cm^−1^ corresponds to C-H_2_ stretching, whereas that at 1638.87 cm^−1^ represents C=O stretching. The band at 1568.94 cm^−1^ is ascribed to N-H stretching, and the band at 1409.99 cm^−1^ corresponds to the C-O stretching of COOH. The band at 1296.85 cm^−1^ indicates the O-H stretching of COOH, whereas those at 1079.79 cm^−1^ and 1041.92 cm^−1^ reflect the O-H stretching of C-OH (Figure 5).

The band at 871.6 cm^−1^ is attributed to N-H stretching in 6-MP [48]. The electron density of the purine ring increased after 6-MP coordinated with Cu^2+^, forming a more stable structure. As a result, the absorption band of the N-H of the purine ring of the HA-DOP-Cu-MP complex was red-shifted to 878.7 cm^−1^. Because of the electron and steric hindrance effects of the copper ions, most of the absorption bands of 6-MP were displaced or disappeared.

#### 3.3.4. XRD Spectra

**Figure 6:**
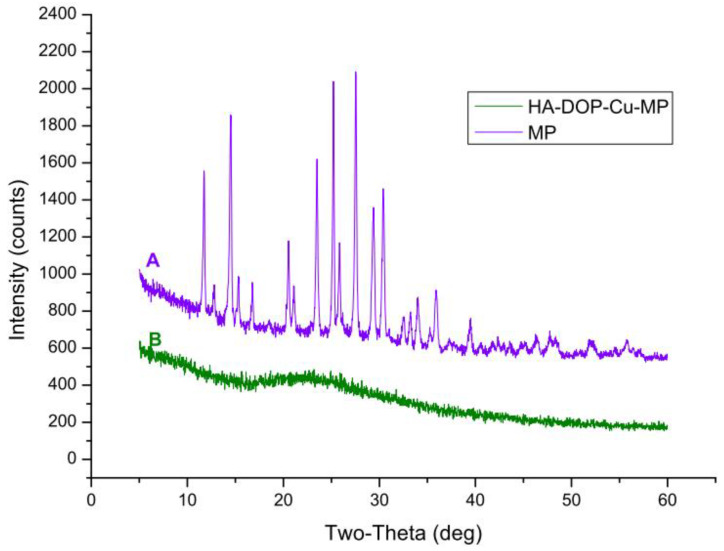
X-ray diffraction (XRD) patterns for (**A**) 6-MP and (**B**) HA-DOP-Cu-MP.

The diffraction pattern for pure 6-MP contained distinct peaks at 2*θ* = 11.7 °C, 14.5 °C, 20.5 °C, 23.5 °C, 25.2 °C, 27.5 °C, 29.4°C, and 30.4 °C. The XRD crystal diffraction pattern (Figure 6A) shows that 6-MP exists as crystals. No characteristic diffraction peaks were found in HA-DOP-Cu-MP (Figure 6B). Indeed, when 6-MP coordinated with HA-DOP and Cu^2+^, it became highly dispersed in the coordination polymer and formed an amorphous structure. Crystalline compounds typically have poor solubility and dissolve slowly, affecting drug absorption and bioavailability. In the HA-DOP-Cu-MP complex, 6-MP exists in an amorphous form, which increases the drug solubility and dissolution rate and is favourable for the dissolution and absorption of the drug.

### 3.4. Drug Release Experiment

Various models have been developed in the field of pharmacy to fit the release behaviour of drugs. The zero-order release rate is constant and unchanging over a period of time, and thus is the ideal release model for controlled release preparations. Drug delivery devices with a zero-order release include osmotic pump and transdermal formulations. First-order release is one of the most common drug release models and consists of the exponential decay of the drug release rate over time. The typical dosage form that follows a first-order release is a water-soluble mesoporous matrix. The Higuchi model is used to fit the skeletal diffusion drug release curve. The Peppas model is an equation that describes dissolution and diffusion and is suitable for polymer drug delivery systems [49].

**Figure 7:**
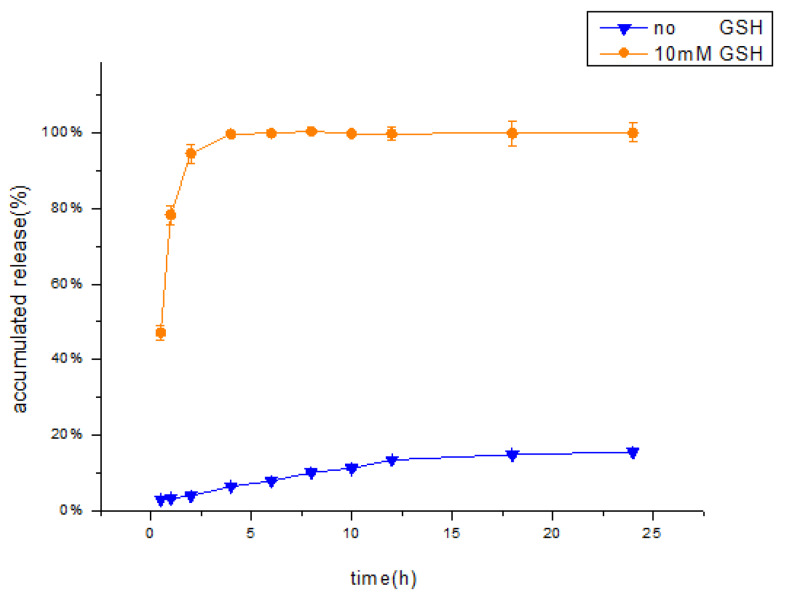
In Vitro cumulative percentage release of MP from HA-DOP-Cu-MP nanoparticles in pH 7.4 phosphate buffer solution (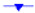 no GSH, 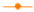
10 mM GSH). The dialysis bag method was used to conduct the release study at 37 °C in a shaker bath (50 rpm).

The data from the in vitro release studies were fitted by various release models [50]. When the release medium contained no GSH, the release profile showed a good fit to the Peppas release model (R = 0.9808) compared with the other models (Table 2), indicating that the HA-DOP-Cu-MP coordination polymer had a good sustained release effect. When the release medium contained 10 mM GSH, the release profile showed a good fit to the first-order release model, indicating that the drug release rate of HA-DOP-Cu-MP was fast, that the drug concentration in the carrier decreased rapidly, and that the release rate exponentially attenuated with time. The in vitro drug release curve is shown in Figure 7.

The influence of GSH on the release behaviour of HA-DOP-Cu-MP was investigated in in vitro release experiments. When 10 mM GSH was added to the release medium, the accumulative release amount of 6-MP exceeded 94% in 2 h. The concentration of GSH in cancer cells exceeded 10 mM, suggesting that HA-DOP-Cu-MP coordination polymer releases 6-MP rapidly to kill cancer cells.

In contrast, in the absence of the GSH, the cumulative release amount was only approximately 15% in 24 h, indicating that HA-DOP-Cu-MP is stable in the systemic circulation. This release characteristic reduces the toxic side effects of drugs to healthy cells.

### 3.5. Cell Uptake Assay

**Figure 8:**
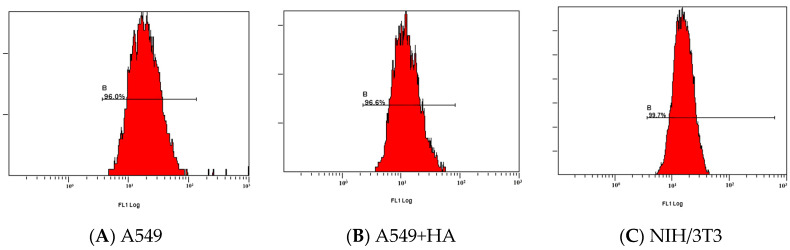
Flow cytometry analysis of the specific endocytosis of fluorescein isothiocyanate isomer I (FITC)-labelled HA internalized by (**A**) A549 cells, (**B**) A549 cells pre-treated for 2 h with free HA (2.5 mg/mL), and (**C**) NIH-3T3 cells.

**Figure 9:**
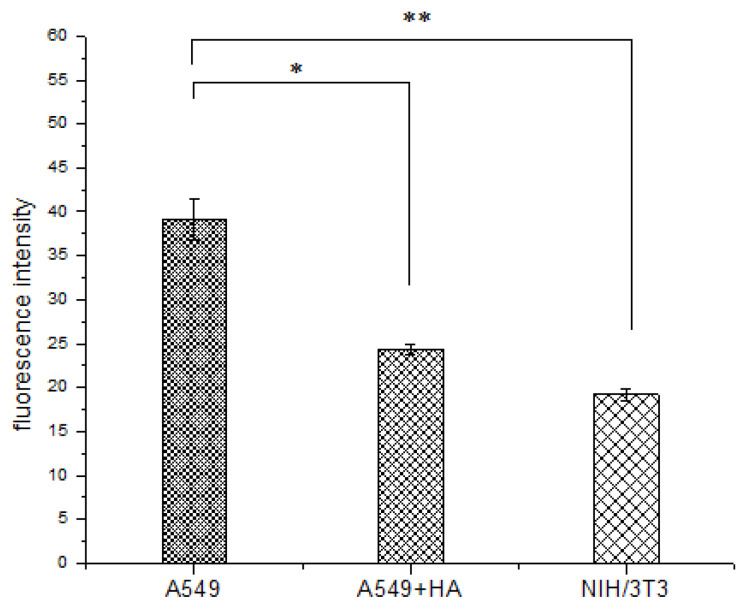
Fluorescence intensity of FITC-labelled HA in A549 cells, A549 cells pre-treated with free HA (A549 + HA), and NIH/3T3 cells. Data are shown as the mean ± standard deviation (SD) (*n* = 3). Statistically significant differences are indicated by * (*p* < 0.05) and ** (*p* < 0.01).

The cellular uptake efficiency of HA-FITC was evaluated by flow cytometry (Figure 8). A549 cells overexpress the CD44 receptor (CD44+) [51]. The fluorescence signal was decreased obviously when A549 cells were pre-treated with free HA (A549 + HA) (Figure 9), confirming that HA competitively inhibited the uptake of HA-FITC by A549 cells. This result demonstrated that HA was internalized by CD44 + A549 cells via receptor mediated endocytosis, indicating that HA could specifically target the CD44 receptor.

NIH/3T3 cells (mouse embryonic fibroblasts cells) were used as CD44-receptor-negative cell lines (CD44−) [52,53]. The fluorescence signal in A549 cells was more intense than that in NIH/3T3 cells (**p < 0.01) (Figure 9), because HA-FITC could only enter the NIH/3T3 cells (CD44−) through nonspecific endocytosis. In contrast, HA-FITC could be rapidly internalized by A549 cells (CD44+) via receptor mediated endocytosis. The cell uptake experiments showed that HA was more readily internalized by cancer cells (A549) than by healthy cells (NIH/3T3). Therefore, this HA drug delivery carrier reduces the toxic side effects of drugs on healthy cells.

### 3.6. Cell Viability Assays

**Figure 10:**
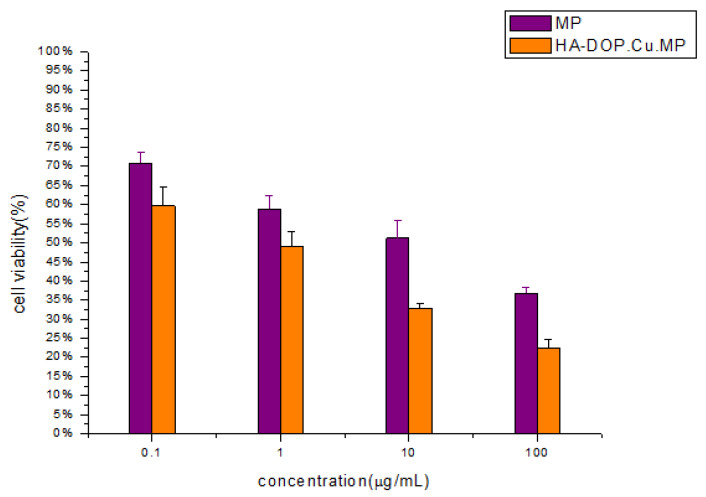
In vitro cytotoxicity of HA-DOP-Cu-MP nanoparticles and 6-MP at various concentrations against A549 cells. Data are shown as the mean ± SD (*n* = 3). The A549 cells were seeded at a density of 4 × 10^3^ cells/well in 96-well microtitre plates. The cell viability of the cells was measured after 48 h by 3-(4,5-dimethylthiazol-2-yl)-2,5-diphenyltetrazolium bromide (MTT) assay. Error bars are based on the SD determined via triplicate experiments (*n* = 3).

The survival rate of human lung adenocarcinoma cells (A549) treated with HA-DOP-Cu-MP and 6-MP decreased as the drug concentration increased after 48 h of administration, and a significant dose–effect relationship was observed (Figure 10). A549 cells overexpress the CD44 receptor and P-gp protein [54]. In tumour cells, the P-gp protein pumps free drugs out of the cell and reduces their cytotoxicity. Nanoparticles are not readily recognized by the efflux pumps after entering cells, and thus they can escape capture by the P-gp protein [55]. In addition, HA nanoparticles are more rapidly absorbed by cells via CD44-mediated endocytosis, and as a result, the cytotoxicity of HA-DOP-Cu-MP to A549 is higher than that of free 6-MP. HA-DOP-Cu-MP is effectively internalized by cancer cells and exhibits good anticancer activity.

## 4. Conclusions

In this study, redox-responsive HA-DOP-Cu-MP coordination polymers were synthesized by a coordination reaction. The synthesis method was simple, and the solubility of 6-MP was improved. The release rate was slow in the release medium without GSH; this release characteristic is favourable for the stability of the drug in the systemic circulation and for reducing the side effects on healthy cells. When the release medium contained 10 mM GSH, the accumulated release amount was approximately 94% in 2 h, indicating that the coordination polymers can quickly release 6-MP in tumour cells containing GSH. After entering the blood circulation, HA-DOP-Cu-MP accumulates in the loose tumor tissue through the EPR effect, and then HA-DOP-Cu-MP can be internalized by tumour cells through CD44-mediated endocytosis. The killing effect of HA-DOP-Cu-MP against tumour cells was better than that of free 6-MP. Indeed, HA-DOP-Cu-MP coordination polymers effectively kill cancer cells and reduce systemic toxicity. Therefore, these targeting coordination polymers have great potential applications in drug delivery for cancer therapy.

Currently, redox-responsive drug delivery systems are typically based on disulfide bonds, which are broken by the reduction of GSH to release drugs. Less research has been focused on coordination polymer drug delivery systems, with most studies addressing pH responsiveness. This work revealed that thiol compounds can be linked to polymers by coordination reactions. The HA-DOP-Cu-MP coordination polymer releases free drugs in a release medium containing GSH and has an excellent redox-responsive targeting. This hyaluronic acid drug delivery system can be used to prepare 6-MP injections. Thus, this study provides a new strategy for the development of redox-responsive drug delivery systems.

## Figures and Tables

**Table 1:** Particle sizes of dopamine-modified hyaluronic acid (HA-DOP)-Cu-MP (*n* = 3).

Sample	Particle Size (nm)	Polydispersity Index (PDI)
HA-DOP-Cu	319.7 ± 25.0	0.529 ± 0.10
HA-DOP-Cu-MP	173.5 ± 5.4	0.363 ± 0.01

**Table 2:** Correlation coefficient obtained by fitting the data for the release of MP from HA-DOP-Cu-MP into a buffered solution at pH 7.4 (with or without 10 mM glutathione (GSH)).

Models	Formula	R (no GSH)	R (10 mM GSH)
Zero-order	M_t_/M_∞_ = k·t + k_0_	0.8968	0.2763
First-order	ln(1 − M_t_/M_∞_)=k·t + k_0_	0.9647	0.9926
Higuchi	M_t_/M_∞_=k·t_1/2_ + k_0_	0.9704	0.4506
Peppas	M_t_/M_∞_ = k·t^n^	0.9808	0.8446
